# Implementing an mhGAP-based training and supervision package to improve healthcare workers’ competencies and access to mental health care in Malawi

**DOI:** 10.1186/s13033-020-00345-y

**Published:** 2020-02-27

**Authors:** Jen Ahrens, Demoubly Kokota, Chitsanzo Mafuta, Mary Konyani, Dennis Chasweka, Owen Mwale, Robert C. Stewart, Madeline Osborn, Blessings Chikasema, Mondie Mcheka, Douglas Blackwood, Sheila Gilfillan

**Affiliations:** 1 Mile End Hospital, Bancroft Road, London, E1 4DG UK; 2 Department of Mental Health, University of Malawi, College of Medicine, P/Bag 360, Chichiri, Blantyre 3, Malawi; 3 Zomba Mental Hospital, Chambo Road, Zomba, Malawi; 4 Malawi College of Health Sciences, Zomba Campus, Zomba, Malawi; 5 Department of Paediatrics, University of Malawi, College of Medicine, P/Bag 360, Chichiri, Blantyre 3, Malawi; 6 Division of Psychiatry, University of Edinburgh, Royal Edinburgh Hospital, Morningside Park, Edinburgh, EH10 5HF UK; 7 Scotland Malawi Mental Health Education Project, Royal Edinburgh Hospital, Morningside Park, Edinburgh, EH10 5HF UK; 8 Royal Edinburgh Hospital, Morningside Park, Edinburgh, EH10 5HF UK

**Keywords:** mhGAP, Malawi, Feasibility, Implementation, Training package

## Abstract

**Background:**

It is now well established that the integration of mental health care into primary care is one of the most effective ways of reducing the substantial treatment gap for mental disorders which exists in most low- and middle-income countries. This study set out to determine whether a Mental Health Gap Action Programme (mhGAP) training and supervision package could be contextualised and implemented within the existing health care system in five districts in Southern Malawi. In addition, the study assessed the feasibility of holding community awareness events and establishing peer support groups in each district to further improve the access of the population to evidence-based mental health care.

**Methods:**

A lead training team of experienced Malawian mental health professionals was appointed and mhGAP training materials were contextualised for use in Malawi. The lead team delivered a 4-day training package to district mental health teams in five districts covering three core conditions: psychosis, moderate-severe depression, and alcohol and substance use disorders. District mental health teams then delivered a 2-day training package and provided monthly supervision for 3 months to 500 non-specialist healthcare workers. Paired sample t-tests were used to compare knowledge, confidence and attitude scores before and immediately after training, and after 6 months in two districts. Case detection rates measured pre- and post-training in the pilot district were compared using Wilcoxon Rank Sum Test. Community awareness events were held and peer support groups were established in each of the five districts. The acceptability of the package was assessed through focus group discussions involving specialist and non-specialist healthcare workers, users and carers.

**Results:**

Non-specialist healthcare workers’ knowledge and confidence scores significantly increased immediately after training in comparison to pre-training. These scores were maintained at 6 months. However, no statistically significant change in attitude scores was detected. Case detection rates increased immediately after the training in comparison to pre-training. Responses from focus group discussion participants illustrated the programme’s acceptability.

**Conclusions:**

This study demonstrated that, with minimal additional funding and working within existing structures, an mhGAP based training at primary and secondary health care levels is feasible in Southern Malawi.

## Background

Although mental health disorders are common in all regions of the world it is estimated that at least 75% of people in low- and middle-income countries (LMICs) do not have access to the treatment they need [1], and the Global Burden of Disease attributable to mental disorder in LMICs is likely to be even greater than previously estimated [2]. The reasons for this treatment gap vary between and within countries and include poverty, especially in rural areas, lack of government funding and a shortage of psychiatrists and other mental health professionals [3]. Mental health services are largely centralised, with specialists based in institutions in the cities [4]. Rural populations are often unaware of treatments available and traditional healers remain the primary source of information and treatment for mental disorders in both rural and urban settings [5]. Enormous social stigma which can affect marriage and work prospects for sufferers and family members is prevalent [6–8].

Minimal access in LMICs to mental health treatments is now universally considered to be one of the Grand Challenges of Global Mental Health [9]. One of the most effective ways of addressing the challenge to reduce the treatment gap is to integrate mental health services into primary care [10, 11]. In 2008, the World Health Organization (WHO) launched the Mental Health Gap Action Programme (mhGAP) designed to scale up care for those with mental, neurological and substance use disorders (MNS). A key stated objective is “to reinforce the commitment of governments, international organisations and other stakeholders to increase the allocation of financial and human resources for care of MNS disorders” [12]. For this to be achieved on the ground in non-specialist settings WHO recommended a “train the trainers model” by which the capacity of primary health care workers to detect and manage mental disorders is improved. The Mental Health Gap Action Programme Intervention Guide for Mental, Neurological and Substance Use Disorders in Non-Specialized Health Settings, (mhGAP-IG) was first published in 2010 [13]. The guide serves both as a teaching and implementation tool, containing guidance on structured assessment and delivery of evidence-based interventions for nine priority conditions: depression, psychosis, bipolar disorder, alcohol and drug use disorders, dementia, developmental and behavioural disorders, epilepsy, self harm/suicide and medically unexplained symptoms. The WHO recommends that the mhGAP-IG is adapted by countries to suit their local context, resources and priorities. Although the guide has now been used widely in a range of contexts, publication on the implementation, impact and effectiveness of mhGAP material within individual country settings, remains limited [14–16].

Malawi is a land locked country in the south east of Africa bordered by Mozambique, Zambia and Tanzania. WHO figures note it had a population of 18 million in 2016 [17] with 50% aged under 16 years [18]. Subsistence farming is the primary source of income for most of its population [19]. In 2014 11.4% of GDP was allocated to health, with no ring-fenced budget for mental health services [17]. Between 2012 and 2015 in excess of 60% of the health budget was externally funded [18].

The health sector is managed centrally by the Ministry of Health. Malawi is divided into Northern, Central and Southern regions, with a central hospital acting as a tertiary referral centre in each. The regions are divided into 28 districts with populations of between 100,000 and 1,000,000, of which half are designated as ‘hard to reach’ [20]. The district health system is the responsibility of the District Health Officer who reports to the District Commissioner, responsible for all public institutions at district level. Each district has a district hospital serving a defined population and taking referrals from health centres and health posts. There is on average one or two doctors per district who work largely in an administrative rather than clinical capacity. The majority of health care is delivered by nursing officers, clinical officers and medical assistants. Population figures, facilities and staffing in each project district are detailed in Table 1.

**Table 1:** Demographic information regarding health services in each district

	Mulanje	Thyolo	Machinga	Nsanje	Ntcheu
Population					
Total	525,429	587,445	488,996	238,099	474,464
Male	247,391	279,979	243,747	115,371	226,567
Female	278,038	307,476	254,249	122,718	247,897
Facilities					
Government hospitals	1	1	1	1	1
Private hospitals	0	0	0	0	0
CHAM hospitals	1	1	0	1	1
Government health centres	18	15	15	10	22
Private health centres	1	20	2	3	4
CHAM health centres	4	8	6	3	13
Number of beds in district hospital	382	298	243	300	255
Staffing					
District hospital (Gov’t and CHAM)					
Doctors	2	2	1	4	3
Clinicians	88	39	48	17	29
Nurses	171	120	76	78	159
Medical assistants	27	49	6	10	47
Psychiatric clinical officers	1	1	0	0	0
Psychiatric nurses	5	4	5	2	2
Health centres (Gov’t and CHAM)					
Nurses	25	57	21	44	16
Medical assistants	20	27	20	20	47

Inpatient mental health services are centralised in one government hospital in Zomba in the Southern region. St John of God Hospitaller Services provide a total of 88 inpatient beds and community services in the Northern and Central regions, based in Mzuzu and Lilongwe respectively. In conjunction with Mzuzu University they also offer training in psychiatry for nurses and clinical officers. Approximately 10–15 clinical officers graduate every 2 years with a 2 year Bachelor of Science degree in Clinical Medicine (Mental Health) awarded by Mzuzu University and St John of God College of Health Sciences. This qualifies them to take up posts as specialist psychiatric clinical officers.

District Mental Health Teams (DMHTs), usually staffed by psychiatric clinical officers and psychiatric nurses, are located in all district hospitals. However, as is common elsewhere in similar settings, staff are regularly taken away from delivering mental health services to cover acute areas such as obstetrics and surgery. Although DMHTs are scheduled to run monthly outreach clinics to health centres in their district these are often cancelled for logistical reasons. There is little integration between mental health and primary care and many primary health care workers do not feel competent to manage those presenting with mental health problems. Shortages and inconsistency of supply of essential psychotropic drugs compound the problems of a lack of human resource and poor infrastructure.

This paper describes the process of country contextualisation and the feasibility of (a) delivering an mhGAP training package to non-specialist healthcare workers (NSHCW) and (b) running community awareness events and establishing peer support groups in five districts in the Southern region of Malawi. We present data on the impact of the training by measuring knowledge, confidence and attitudes scores, and the detection rate of mental disorders. In addition, we present qualitative data from focus group discussions which were held in all five districts. Although all aspects of the design and implementation of the project were decided following extensive consultation with the Ministry of Health (MOH) and the College of Medicine in Malawi, it was not part of a formal government programme.

## Methods

### Appointment of in-country staff

A lead training team (LTT) of three experienced mental health clinicians; a psychiatric clinical officer, a nurse lecturer at the Malawi College of Health Sciences, and a psychiatric nurse, was appointed after competitive interview. The College of Medicine (COM), Blantyre, Malawi was responsible for the interview process which was conducted as per College protocols. Posts were advertised in local and national newspapers and on the College’s website. The Head of Department of Mental Health (JA) and Visiting Lecturer (SG) took part in both the selection and interview process. Each individual appointed had extensive experience of working within the healthcare system in Malawi, and of teaching and training in mental health. One trainer left for personal reasons 6 months into the project was replaced by three additional trainers (two psychiatric clinical officers and one psychiatric nurse) working part time on flexible contracts. The LTT was supported by the Head of Department (JA), Visiting Lecturer (SG) and a half time project manager (DK) in the College of Medicine.

### Selection of districts

The five districts, Mulanje, Thyolo, Machinga, Nsanje (Southern Region) and Ntcheu (Central Region) were selected following discussion with the Ministry of Health (MOH) and on the basis that each had a psychiatric clinical officer in post at the time they were identified. Although covering a wide geographical area spread over the Southern region the districts were all deemed to be accessible within the time frame of the project. District Health Officers (DHO) had been asked by the MOH to participate and all had agreed to do so.

### Adapting mhGAP for use in Malawi

The initial task was to adapt mhGAP training materials for the country and project contexts. Permission was gained from WHO to adapt field test versions of the teaching materials for base and standard courses. The study was conducted prior to the publication of WHO guidance on country and context adaptation which is now readily available. Grant funding was insufficient to support the teaching of all mhGAP conditions. It was agreed following discussions with the MOH during the grant writing process that training would be limited to the core conditions of psychosis, moderate-severe depression, and alcohol and substance use disorder. It was acknowledged that these serious mental illnesses are amongst those neuropsychiatric conditions which account for the greatest burden of disease attributable to mental disorder [21].

A series of discussions took place involving the LTT and SG. JA and others with experience of delivering a cascade model of training in mental health in low-income countries contributed to these discussions. The main challenge in adapting the training materials was to shorten them to develop a 4 day training for DMHT staff and a 2 day training which they could then deliver to NSHCW, while ensuring that all essential components of the modules were taught. To achieve this we maintained the structure of Introduction followed by Learning Objectives, Key Actions, Plan and Start Management, Link with Other Services and Supports, Follow Up and Key Messages for each condition.

The adaptation included the use of an existing series of videos prepared for teaching medical students in Chichewa, the language spoken in the five districts, and adaptation of role plays and case vignettes for local use. Handouts and power-point presentations were similarly adapted. Risk assessment, focussing on risk of suicide, was included in each module. The Mental Health Users and Carers Association (MeHUCA), a nationally registered patient advocacy organisation, worked with the LTT to develop a presentation used in the training of both DMHTs and non-specialist healthcare workers. The mhGAP Training of Trainers and Training of Supervisors modules were likewise adapted and shortened into two 2 day trainings for DMHT staff.

Updated Malawi Standard Treatment Guidelines, including for mental health, were published by the MOH in during the project roll out and incorporated into the training [22].

It was apparent at an early stage that the use of the mhGAP-IG would not be appropriate in the primary care setting in Malawi for a number of reasons. The DMHTs who were to become trainers were clinical officers and nurses who had a very variable range of skills and experience. Most had undergone mental health training many years previously with no refresher training, and were not working solely in mental health. The duration of training by the LTT for which the project was funded was insufficient to ensure that the DMHT could effectively use the Intervention Guide.

### Delivery of the training

At least two preliminary visits were made to each district by members of the LTT, project manager and JA/SG to meet with the District Health Officer, DMHT, clinical co-ordinator, chief nurse and pharmacist. The LTT delivered a 4-day training to each DMHT covering the core conditions of psychosis, moderate-severe depression and alcohol and substance use disorder. Approximately 6 weeks after the initial training the LTT delivered a 2-day training on teaching and after approximately 8 weeks, 2 further days on supervision.

The NSHCW trainings were organised by the DMHTs to ensure that clinical service was not compromised. Staff from different locations were trained in groups of approximately 25 ensuring adequate staffing remained at the health centres and clinical areas. In Mulanje, the pilot district, non-specialist primary health care workers in all government run health centres were trained. This model was revised for subsequent districts as all non-specialist staff rotate between the district hospitals and health centres and there was a demand from staff based in the district hospitals for training. The training comprised a 2-day condensed version of the 4-day training.

### Supervision

It had been planned to deliver supervision once a month for 3 months post-training via the mobile outreach clinics and to include direct clinical observation of NSHCW assessing patients presenting with mental health problems. Experience in the pilot district, Mulanje, showed that this was not possible for logistical reasons. Even when funds were made available to ensure that the outreach clinic went to all health centres on a monthly basis, staff were often too busy to take part in supervision. The decision was made to bring staff back for monthly supervision to a central location in the same groups in which they had been trained. Liaison with the district clinical co-ordinators ensured that health centres were not left unstaffed during supervision sessions and the arrangement worked well. Supervision was primarily delivered through case-based discussion. NSHCW were encouraged to make notes about individuals whom they had seen in the course of their work during the previous month and bring these to supervision. This was supplemented by group discussion using clinical scenarios on occasion when participants did not bring sufficient cases to supervision.

### Community awareness events

In parallel with the training, community awareness events were held in each district. The project manager, a project assistant and members of MeHUCA made at least two planning visits to each district. Senior district hospital management staff and DMHTs identified preferred locations based on high numbers of individuals known to have mental health problems in the area and, in one case, because of a well-known user of services with a powerful recovery story. Discussions took place with village chiefs, religious leaders, head teachers, health education officers, health centre staff and local service users. Each event was well advertised with banners and drive by megaphone the day before and local journalists were invited to attend. The purpose of the event was to educate the community about mental illness, services available and the importance of community support for individuals. Senior chiefs played an important role in each event. Staff from the DMHTs and health centres spoke at each event as did members of MeHUCA and local service users. The message was reinforced with performances by local dance, drama and musical groups.

### Peer support groups

Members of MeHUCA working with the project assistant (DC) established peer support groups in each district. This was done through a combination of the trainings, publicity at the community awareness events and links made between MeHUCA members and service users and carers attending clinics at the district hospitals. Existing MeHUCA members and the project assistant were able to offer initial support to the new groups, as was the DMHT.

### Programme evaluation

#### Knowledge, confidence and attitudes

Self-report knowledge, confidence and attitudes questionnaires were distributed to all participants by the trainers at the first and last trainings, and returned on the same day. In the first and last districts trained, Mulanje and Ntcheu, they were also administered at 6-month follow up. Questionnaires were in English with all participants being fluent. For budgetary reasons we could not collect 6-month follow up data in all districts.

Knowledge scores about the identification and management of mental disorders were obtained using a pre- and post-knowledge test adapted from an earlier 20 question version of mhGAP online training materials to exclude questions about conditions not taught [23]. The adapted version consisted of 6 true/false and 9 multiple choice questions. The pre- and post-knowledge questionnaire has been used to evaluate a number of mhGAP interventions [24–28].

Confidence scores were obtained with a questionnaire used in a similar project in Malawi [29] in which the capacity of health surveillance assistants to recognise and respond to individuals with both common and severe mental health problems was scaled up. The tool was developed specifically for that project. Participants rated themselves on 14 confidence questions using a 4-point Likert scale from 1 (very confident) to 4 (not at all confident).

Attitude scores were obtained using the Community Attitudes to the Mentally Ill Scale [30]. It has been demonstrated to be reliable in and has been used in other sub-Saharan populations [31–33]. The four subscales (benevolence, authoritarianism, community mental health ideology and social restrictiveness) each have 10 questions rated on a 5-point Likert scale. These were combined to give a total attitudes score.

#### Data analysis

The distribution of all variables were checked before conducting any analysis using Stata version 13.1 (Stata Corp, Texas, USA). For continuous variables, summary statistics were obtained and presented either as a median (inter quartile range [IQR]) or mean (standard deviation [SD]) dependent on whether the data was normally distributed.

Given that the scores for the variables knowledge, confidence and attitudes were normally distributed, a paired t-test was conducted to assess any change in these measures after training.

#### Case detection

Case detection rates were available for 6 months (Jan–Jun) pre- and 6 months (Jul–Dec) post-training in 2014 for the pilot district, Mulanje. Case detection rates were ascertained by inspection of the hand-written case registers held in each of the 18 health centres for all attendances by the project manager (DK). Mental health diagnoses were restricted for coding purposes to acute and chronic psychosis and epilepsy. Median number of cases detected per month in the 6 months pre- and post-training were compared using the non-parametric Wilcoxon Rank Sum Test.

#### Focus group discussions

Four focus group discussions (FGDs) were held in each district in the 6 months after training. One each with the DMHT and NSHCW, and two with users and carers. One user and carers discussion group took place at the mental health clinic at the district hospital, and the other at a busy health centre mental health clinic. The FGDs were led by a research assistant with experience of conducting FGDs in mental health research in Malawi who had not been involved in the training, and who had been employed specifically for this role. Each DMHT was invited to attend a focus group and they in turn invited 10–12 NSHCW to attend a separate FGD. Users and carers were selected from those attending the clinic on the day of the FGD. The lead clinician at the clinic was asked to suggest 8–10 people who were currently well enough to participate or were a suitable carer. All potential participants gave written consent to participate. FGDs lasted up to an hour, followed a structure, were recorded, transcribed and translated where necessary into English.

Unfortunately during the process of transcription and translation, the recording devices and laptop with the FGD recordings were stolen. No back up copy had been kept and this prevented formal qualitative analysis of the content of the FGDs. Thematic analysis of the remaining material was conducted by the project lead (JA) in order to make use of the rich material obtained. We have presented the themes and supporting quotes.

## Results

### Training of non-specialist health care workers

Supported by the LTT, DMHTs trained and supervised 500 non-specialist health care workers working in primary care and district hospitals. A total of 27 two-day trainings were delivered by 5 DMHTs and all participants were supervised monthly for 3 months post-training. All districts were similar in terms of demographic makeup (Table 1).

We achieved near total coverage of NSHCW trained in each district. All NSHCW were invited to attend training with less than 20 workers across all five districts failing to respond and therefore not receiving training. We ran the training 4–7 times in total per district to maximise the opportunity for workers to attend. In the supervision sessions across all five districts, 78% attended the first round of supervision, 60% the second and 62% the third.

Of the 500 participants 51% (255) were male. Fifty-two per cent (259) worked as a nurse/midwife/technician with 21% (107) working as medical assistants. The median age of NSHCW was 30 years with a median of 5 years clinical experience. Only 12% had previous mental health training (Table 2).

**Table 2:** Baseline characteristics of study participants by district

	Mulanje	Thyolo	Machinga	Nsanje	Ntcheu
Gender n (%)					
Male	26 (60.5)	35 (46.1)	59 (46.1)	73 (57.9)	62 (48.8)
Age in years					
Median (IQR)	34 (30–42)	30 (27–44)	28 (25–34)	31 (27–36)	31 (27–38)
Current role n (%)					
Nurse/Midwife/Technician	19 (44.2)	28 (31.6)	70 (54.7)	65 (51.6)	77 (61.1)
Medical assistant	20 (46.5)	21 (27.6)	27 (21.1)	21 (16.7)	37 (29.3)
Clinical technician	1 (2.3)	13 (17.1)	22 (17.2)	29 (23.0)	5 (4.0)
Community nurse	2 (4.7)	4 (5.3)	5 (3.9)	11 (8.7)	2 (1.6)
Other	1 (2.3)	10 (13.1)	4 (3.1)	0	5 (4.0)
Years of clinical experience					
Median (IQR)	5 (3–10)	6 (4–10)	4 (0.7–8)	5 (2–10)	5 (2–10)
Previous training in mental health n (%)					
No training	40 (93.0)	59 (79.7)	113 (88.3)	116 (92.1)	108 (87.8)
In-service training in mental health n (%)					
No training	43 (100)	66 (86.8)	124 (99.2)	125 (99.2)	123 (97.6)

Overall 499 (99.8%) completed pre- and post-training questionnaires. In the two districts, Mulanje and Ntcheu, in which 6-month post-training data were collected 122/170 (71.8%) completed all three pre, post and 6-month questionnaires.

#### Knowledge

The overall (n = 499) mean knowledge score increased from 11.8 (SD 2.52) pre-training to 14.5 (SD 2.63) immediately post-training [paired t-test, p < 0.001 (Table 3)]. In Mulanje and Ntcheu districts (n = 122) mean knowledge score remained increased at 13.5 (SD 2.08) 6 months post-training [pre-training versus 6 months post-training paired t-test, p < 0.01 (Table 4)].

**Table 3:** Knowledge, confidence and attitudes

	n	Knowledge			Confidence			Attitudes		
		Pre	Post	Paired t-test	Pre	Post	Paired t-test	Pre	Post	Paired t-test
		Mean (SD)	Mean (SD)	p-value	Mean (SD)	Mean (SD)	p-value	Mean (SD)	Mean (SD)	p-value
Mulanje	43	11.8 (2.16)	15.1 (2.53)	< 0.001	36.9 (7.69)	49.6 (6.14)	< 0.001	127.3 (8.93)	128.9 (10.40)	0.45
Thyolo	76	12.3 (2.72)	13.9 (2.70)	< 0.001	38.6 (9.39)	46.5 (8.86)	< 0.001	121.3 (12.12)	121.5 (12.82)	0.99
Machinga	128	12.0 (2.74)	14.8 (2.87)	< 0.001	35.0 (8.52)	47.2 (8.20)	< 0.001	121.2 (11.35)	120.2 (11.48)	0.29
Nsanje	126	11.8 (2.35)	14.7 (2.87)	< 0.001	35.8 (9.89)	46.5 (8.86)	< 0.001	122.6 (11.17)	121.3 (8.88)	0.25
Ntcheu	127	11.3 (2.38)	14.0 (2.63)	< 0.001	37.0 (8.06)	49.6 (6.14)	< 0.001	118.3 (14.52)	121.8 (16.50)	0.56

Distribution of mean knowledge, confidence and attitudes scores, pre- and post-training, by district

**Table 4:** Knowledge, confidence and attitudes

	n	Knowledge		Confidence		Attitudes	
			Paired		Paired		Paired
		6 months	t-test	6 months	t-test	6 months	t-test
		Mean (SD)	p-value	Mean (SD)	p-value	Mean (SD)	p-value
Mulanje	43	13.9 (2.52)	< 0.01	46.8 (6.03)	< 0.01	128.9 (10.4)	0.55
Ntcheu	79	13.2 (1.92)	< 0.01	43.9 (4.85)	< 0.01	122.5 (12.1)	0.06
Combined	122	13.5 (2.08)	< 0.01	45.4 (4.71)	< 0.01	124.1 (11.7)	0.29

Distribution of mean knowledge, confidence and attitudes scores at 6 months post-training, by district and combined

#### Confidence

The overall (n = 499) mean confidence score increased from 36.6 (SD 8.6) pre-training to 47.8 (SD 7.3) immediately post-training [paired t-test, p < 0.001 (Table 3)]. In Mulanje and Ntcheu districts (n = 122) mean confidence score remained increased at 45.4 (SD 4.71) 6 months post-training [pre-training versus 6 months post-training paired t-test, p < 0.01 (Table 4)].

#### Attitudes

There was no difference in the overall mean score on the CAMI from pre-training to immediately post-training (Table 3) or between pre-training and at 6 months post-training in Mulanje and Ntcheu districts (Table 4).

#### Case detection rates

In the 6 months (Jan–Jun) before training the median number of cases per month from all 18 health centres in Mulanje was 77 (IQR: 65–87) whereas in months after training (Jul–Dec) the median number of cases was 186 (IQR: 175–197) showing a significant increase in median number of cases before and after the training; p-value < 0.001 (Wilcoxon Rank Sum Test).

#### Thematic analysis from focus group discussions

There were a number of themes that emerged from the focus group discussions.

On the whole the training was seen as of value and of interest to the NSHCWs. Mental health care was an area of work that the NSHCWs had felt ill equipped to undertake, however the training package had helped them to develop the skills to feel more confident in initial assessment and management. Almost universally, stigmatising attitudes towards people with mental disorders were expressed by the NSHCWs as a potential barrier to care.

NSHCW Mulanje:

“In the past if someone comes with a psyche patient, that’s how we call them, there was no taking of the patient’s history or doing anything at all, we would just refer that patient to this hospital but now we know two or three things that you may be able to ask and options of things that you can do in order to help this patient. That is how I understand it”

“Let me put it like this, in the beginning, we as health workers thought that there is no possible way of helping these people or that they would not even be able to sit amongst people but after the training we were given the opportunity to learn that these people are just as human as every patient that has come to the health facility therefore he has rights to be helped as everyone else”

“But also, here in Mulanje, we have many people with this condition perhaps it is because of smoking weed or alcohol consumption, we don’t know. So because of this, we wouldn’t consider these people as important and we would criticize them, be wicked towards them to an extent of locking them inside our homes because they are being troublesome so during the training we learnt that if the patient has ‘’psyche’’ problem, this person is also in need of our attention because there is definitely something wrong with him. The first thing is to take our time with him, if he has been tied we need to tell the guardians to untie him and then we should welcome him, then take his history as to what happened. So in this regard we feel things are going well now”

The training package had an impact on the practice of the NSHCWs at the mental health clinics and also on the experience of the Users and Carers attending the clinics.

DMHT Mulanje:

“When you go the facility, there are other individuals you can see they are really practicing because you can see by the case they are presenting when you had gone for supervision, you could see somebody presenting a case knowing the case in and out and even the management so I feel mostly some people have gained and are able to see mental health problems”

“We have seen a change in the sense that most of the health passport books, mostly as he said, they were just writing referral to Mulanje district hospital but now they are able to put the complaints of the patient and the management is there even in the notes they are able to write the signs and symptoms, the diagnosis, any other physical investigations they have done and then the management with the follow up, yes some health care workers are able to do that”

User and Carer Thyolo:

“Let me just add on to what he has just said. I can differentiate with the way things were before. We would get shouted at amongst other things but now we have seen a change. We have noticed that they are giving us enough medicine.”

“I am able to see positive change because I am able to come and get assistance here. I was unable to come here in the past, there was no proper counselling. Right now because of the advice given to us and followed, I am able to see that we are being assisted”

“I feel that ever since I started taking this medication things have changed. Furthermore, I am able to do my work such do farming and other things. I feel things are well as compared to the way things were before.”

However a number of challenges regarding implementation of the training emerged:

The allocated remuneration to cover food, transport and accommodation was deemed to be insufficient and there were significant logistical challenges for participants in attending the training in terms of the distance travelled and time required.

DMHT Mulanje:

“also the allowances given to the participants, they were different in terms of…they were saying that those who live nearby will not be given and those staying far will be given night and that demotivated the participants and some did not even participate properly. They said that we will not do this work well, when you are coming we will be running away and not give you data or help you”

NSHCW Mulanje:

“yes, the money was not enough when you compare it to where we were coming from and we would wake up at three, four in the morning to come here”

“I think that the logistics was not very good and some of us come from far and with the way it was organized I feel that they did not consider us very much in terms of how we would travel”

The participants and the trainers thought that there was insufficient time to cover the material presented and that additional follow up training would be of benefit.

DMHT Mulanje:

“Another challenge is on how they designed the training; the period of the training….it was compressed. It was like an orientation, just a few days and the content was a lot and it needed a number of days so we were teaching against time”

NSHCW Mulanje:

“I want to say that psyche is a lot and the days in which the training was conducted were not enough…….. there are still some things of which we are still in doubt over, we can ask ourselves, is this mania or what, we are in doubt so I would have loved if we had a chance to go the ward for practicals”

There were logistical barriers at the health centre clinics in implementing the training, given the high work load and limited time available to the NSHCW at the primary care clinics. The lack of availability of psychotropic medication at the primary care clinics could lead to people being referred to the district hospital even if the NSHCW undertook the assessment.

NSHCW Mulanje:

“what I have noticed is that these people need more time which we don’t have at the facility…more time in consultation because when we have a patient explaining their history, its comes out straight forward but others it doesn’t come out like that”

“and also to add to that, we never used to have the medicines at the health centers even if we order but now they give us so they are benefiting now unless we really need to refer them maybe because we don’t have the medicine”

#### Community awareness events

Community awareness events were held in each district and were attended by between 300 and 700 people.

## Discussion

Using an mhGAP training package adapted for the Malawian context we have demonstrated an improvement in the knowledge and confidence of 500 non-specialist healthcare workers in the recognition and treatment of core mental disorders.

Initially we had concern that the high workloads already placed on non-specialist healthcare workers in district hospitals and primary care clinics coupled with the stigma related to mental health, would be barriers to the successful implementation of the training. However, we found that the majority of participants were attentive and engaged well in the interactive training process and subsequent supervision. The numbers that participated in both the training and supervision would support this. Other mhGAP programmes have struggled with recruitment and retention of participants with lack of interest cited as a cause [27]. While there was some loss of NSHCW attending supervision, a majority attended each of the 3 monthly sessions, despite significant logistical barriers and dissatisfaction about the allocated rate of remuneration for expenses. Almost all NSHCW in the districts were trained, without any selection being used to target those most interested in mental health [26].

We found that although the training model relies on a ‘cascade’ of training, from the LTT to DMHTs and then to non-specialist workers, the DMHTs were not able to deliver the 2-day package independently. They required support to do so to ensure that the quality of the training was maintained, which has been a concern in other programs where a ‘train the trainers’ model is used [34]. As the training was rolled out it was necessary for members of the LTT to support the DMHTs to deliver both training and supervision. In particular they required support in using interactive teaching methods and audio-visual equipment.

However, using this model undoubtedly strengthened professional relationships between staff working in district hospitals and those in primary care. It highlighted the role of the DMHT as experts available for consultation about difficult cases. Likewise, the relationships between the LTT, with their affiliations to the mental health hospital, and district hospital staff strengthened. Anecdotally clinical staff in the mental health hospital reported an increase in telephone consultations from DMHTs with a decrease in inappropriate admissions. This strengthening of relationships has been suggested as a factor in the success of programmes to develop mental health services in low resource settings [35] in which primary care models need to be “sustained by integrated, functional and mutually supportive referral systems”.

With the engagement and support of the MOH from the outset we were able to have ongoing discussions with the Ministry and DHOs. It was possible to highlight in some detail the challenges of maintaining a reliable supply chain of psychotropic medications and to explore ways of addressing these. Although at the end of the project the MOH had no budgetary allocation to fund a widespread roll out of the training package, there was considerable interest in attempting to incorporate it into other externally funded programmes.

A systematic review of the implementation and evaluation of the mhGAP-IG highlights the need for reporting contextual strengths and challenges to implementation [36]. The many logistical barriers to delivery of this training to 500 healthcare workers cannot be overstated. To attend the training and supervision many healthcare workers had to travel more than 100 km from health posts in remote areas. There were floods and travel in rainy season was particularly difficult. Many of the training facilities had periods of time without electricity so that audio-visual aids for teaching could not be relied upon.

All of the activities of the project were conducted over a 3 year period at a cost of approximately £125,000. The project was one component of a larger programme funded by the Scottish Government through a Malawi Development Grant and administered through COM. It included the salaries of the LTT and one lecturer’s post for 6 months, the training and supervision costs including stipends to cover transport, food and accommodation for all participants, and costs for the community awareness raising events. It did not include indirect costs such as the NSHCW time away from post to attend training etc. The direct costs of delivering the training to DMHT and NSHCW, together with supervision and running community awareness events, was approximately £15,000 per district. As this was funded as part of a development grant there was no budget to support roll out of the training package. It is difficult to make comparisons with other similar trainings, as project costings are rarely reported in the literature. Only 3 of the 33 papers included in a recent systematic review included economic evaluations [36]. Estimating the true total cost is complex, with consideration of direct and indirect costs needed. Input from a health economist is unlikely to be readily available in small development projects such as ours. Financial investment is a key aspect in the discussion with funding bodies or governmental agencies when consideration is given to widespread roll out. Sustainability of such programmes is a cause for concern in global mental health research, with implementation science now starting to address the knowledge gap. Demonstrating value and effectiveness of mhGAP training programmes will help to inform this debate.

### Strengths

The use of Malawian mental health experts, with their deep knowledge of the local context and concerns, as lead trainers, gave the training credibility compared to employing international experts. Although the training materials were mainly in English, the fact that both the LTT and DMHT were local allowed more nuanced expression and discussion in the local language. We believe that this also engendered a sense of ownership of the trainings by the mental health staff in each district.

Several preliminary meetings were held with hospital management and clinical staff in each district and we believe formed the basis of a respectful relationship and communication style that continued throughout. This together with the use of local staff, support from the Ministry of Health and institutional leadership from the College of Medicine lent the project authority and were key factors ensuring the smooth running of the project and high uptake of training by non-specialist health care workers. It was helpful that there had been prior agreement to implement the project between the MOH and the DHOs and we were grateful for their continued support. Significant engagement and consultation with all levels of health service administration, prior to commencement, is considered to be a factor in successful implementation of such programmes [10]. All of the discussions which took place during the development of the training package and prior to its implementation allowed us to address, at least some of, the cultural and contextual challenges which have been identified as presenting major barriers to mhGAP implementation [37].

Supervision was a key element in the success of the project and strengthened the knowledge gained during training, giving a forum for peer to peer discussion about cases as well as feedback from trainers. We were quickly able to adapt to deliver supervision in central locations without compromising delivery of health care in health centres. Training programmes which have supervision included are known to have greater impact on health care provider practices in low income settings than training alone [38]. In an evaluation of mhGAP in Fiji, lack of supervision was cited as a significant barrier to the implementation of mhGAP training in practice [34].

The training was inclusive, involving service users through direct involvement and through screening a film made in Malawi and previously shown on the national television channel documenting a service user’s experience of a psychotic illness and recovery. In parallel we were able to engage the community in awareness raising events and establish users and carers groups. Advocacy and engagement with stakeholders in the community are key to improving mental health services in LMICs. Saraceno et al. in the 2007 Lancet Series on Global Mental Health state that ‘Non-formal community resources will need to be recognised and mobilised to ensure access to care for the millions of people who need it’ [35].

### Limitations

Piloting use of the questionnaires used to assess knowledge, confidence and attitudes to assess reliability and validity in the study population would have strengthened confidence in the evaluation of the training. This was not feasible due to budgetary constraints.

The use of a test retest design to evaluate the programme is open to potential information bias, as test retest reliability is needed to address concerns about the impact of other factors accounting for the change seen over time. This limits the strength of the conclusions drawn from our evaluation.

Selection bias in the selection of focus group discussion participants is a well recognised methodological concern in their use and was not fully addressed in our study.

While case detection rate was measured in one district, there was no objective assessment of impact on delivery of care or clinical outcomes of service users in the five districts.

The operational research approach and limited budget meant that outcome data at 6 months was only available from two districts. We believe that the retention of knowledge and confidence at 6 months is likely to be similar in the other three districts, given the similarity in the demographics and immediate pre- and post-test results in all of the districts.

The evidence of increased case detection in the 6 months post-training was only available from the pilot district. Again while we believe that it is likely that case detection rates increased in the other districts, we cannot extrapolate.

Case detection rate was assessed from hand written case registers, and may have resulted in information bias. Having said this although there was an electronic health management information system at district level this was dependent on the inputting of hard copy data from health centres which was inconsistently sent to the district hospital and inputted.

While we have demonstrated that it is feasible to contextualise and implement an mhGAP training package, demonstrating an improvement in the participants’ knowledge and confidence scores in recognition and treatment, we found no change in their attitudes towards people with mental disorders. We know that attitudes are more resistant to change than other outcome measures [39] and it is possible that had we been able to repeat the CAMI at 12 months, change may have been evident.

## Conclusions

1. The findings of this study indicate that a 2-day mhGAP training package adapted for use in Malawi can be delivered to large numbers of non-specialist health care workers, and the improvements in knowledge, confidence scores and case detection rate of mental disorder in our sample may be useful in informing further research.
2. Supervision can be provided at low cost and without disruption to clinical services by providing this at central locations.
3. Using a “train the trainers” model may ensure that the knowledge and skills of district mental health teams are updated and strengthened, remaining as a resource within the district.

This training in mental health was acceptable to district health management staff, district mental health teams, non-specialist health care workers, and users and carers.

This study has demonstrated the feasibility and acceptability of using an mhGAP training package to improve access to evidence based mental health care at primary and secondary health care levels in a significantly resource constrained setting within existing structures, and with minimal additional funding. We believe that this training package can be rolled out to the remaining 23 districts in Malawi, and that our experience and lessons learnt can help to inform mhGAP training programmes in other resource constrained LMIC settings.

## Data Availability

The datasets used and analysed during the current study are available from the corresponding author on reasonable request.

## Abbreviations

CAMI: Community Attitudes Towards the Mentally Ill
CHAM: Christian Health Association of Malawi
COM: College of Medicine
DHO: District Health Officer
DMHT: District Mental Health Team
FGDs: Focus group discussions
LMIC: Low- and middle-income countries
LTT: Lead training team
MeHUCA: Mental Health Users and Carers Association
mhGAP: Mental Health Gap Action Programme
mhGAP-IG: Mental Health Gap Action Programme-Intervention Guide
MNS: Mental Neurological and Substance Use Disorders
MOH: Ministry of Health
NSHCW: Non-specialist Health Care Workers
SD: Standard Deviation
UCT: University of Cape Town
WHO: World Health Organization
